# Antibiotics and Non-Targeted Metabolite Residues Detection as a Comprehensive Approach toward Food Safety in Raw Milk

**DOI:** 10.3390/foods10030544

**Published:** 2021-03-06

**Authors:** Luca Maria Chiesa, Federica Di Cesare, Maria Nobile, Roberto Villa, Lucia Decastelli, Francesca Martucci, Mauro Fontana, Radmila Pavlovic, Francesco Arioli, Sara Panseri

**Affiliations:** 1 Department of Health, Animal Science and Food Safety, University of Milan, Via Celoria 10, 20133 Milan, Italy; luca.chiesa@unimi.it (L.M.C.); federica.dicesare@unimi.it (F.D.C.); roberto.villa@unimi.it (R.V.); radmila.pavlovic1@unimi.it (R.P.); francesco.arioli@unimi.it (F.A.); sara.panseri@unimi.it (S.P.); 2 Istituto Zooprofilattico Sperimentale Piemonte, Liguria e Valle d’Aosta, Via Bologna, 148, 10154 Turin, Italy; lucia.decastelli@izsto.it (L.D.); francesca.martucci@izsto.it (F.M.); 3 DVM, Specialist in Inspection of Food of Animal Origin, 10147 Turin, Italy; medivet2@gmail.com

**Keywords:** antibiotics, metabolites, non-targeted analysis, Compound Discoverer, milk, screening test, HPLC-HRMS, food safety

## Abstract

Antibiotic residues in milk are a serious health and technological problem in dairy processes. This study aims to verify the absence of administered antimicrobials after therapeutic treatments, taking into consideration the withdrawal period, and to evaluate the reliability of screening tests under field conditions after confirmatory HPLC-HRMS (High Performance Liquid Chromatography-High-Resolution Mass Spectrometry) Orbitrap analysis. Moreover, the presence of expected or non-targeted metabolites was investigated using the new Compound Discoverer approach. The presence of antimicrobial drugs was shown in 29% of the samples, and also sometimes their metabolites (for enrofloxacin and lincomycin), despite the fact that samples were collected at the seventh milking. Moreover, in 9% of the samples, undeclared treatments were revealed due to the presence of both parent drugs and metabolites. Lastly, the putative identification of two new enrofloxacin metabolites, ENRO-N-methylacetamide and ENRO-ornithine, was proposed. In the light of this evidence, it must be borne in mind that metabolites, some of which are pharmacologically active, may also pose a risk to consumers and for the entire processing of milk in the cheese industries.

## 1. Introduction

Antibiotics have been widely used in animal husbandry for over 60 years for the prevention of and therapy for common pathologies (mastitis, respiratory and podal diseases, neonatal diarrhea, etc.) and prophylactic purposes [1]. Moreover, misuse of antibiotics to increase growth performance and feed efficiency, or to synchronize and/or control the reproductive cycle and breeding performance [2], can lead to the presence of antibiotic residues in milk, a worrying issue for public health that requires investigation.

Concerns over antibiotic residues in food of animal origin arise due to the potential threat of direct toxicity to consumers, but mainly because low dosages of antibiotics could result in the alteration and possible development of resistant strains of bacteria and the consequent failure of clinical antibiotic therapy [3].

Regarding the above-mentioned main uses, the presence of residues in milk may be due to the miscellaneous use of antibiotics, either directly (e.g., administration of drugs to animals) or indirectly (e.g., from the farming and production environments), representing a threatening issue for consumer health [4]. In particular, among the indirect sources of contamination, the most important could arise from air and water during the processing, storage and transportation of milk and dairy products, the improper cleaning of antibiotic contaminated equipment or improper disposal of empty containers of antibiotics in the farm, which could contaminate feed or feed given to animals [5]. There are many causes of miscellaneous use, from inadequate information supplied by manufacturers, lack of awareness, lack of safer drugs and misuse to increase production and profit from animals. If farmers do not follow the instructions supplied with antibiotics correctly, residues of antibiotics may be found in milk. Furthermore, when an antibiotic is indicated as being only for human therapy, their use in animals is ill-advised. Use of antibiotics in different species, under conditions for which they have not been approved or in amounts higher than the prescribed concentration, is to be considered extra-label use [2]. Another main cause of the presence of antibiotic residues in milk may be the lack of a proper withdrawal period in cows. Full responsibility of the veterinarians and farmers in observing the withdrawal period of a drug prior to slaughter is essential to avoid high or illicit antibiotic residue concentrations in milk. Often, to circumvent checks, small doses of drugs are used in cocktails for a synergistic effect. Furthermore, the presence of antibiotic residues in milk, even in low concentrations, can interfere with fermentation during technological cheese-making processes by inhibiting the starter culture, as was demonstrated in our previous work [6]. On the basis of these premises, despite the fact there are MRLs (maximum residue limits) for antibiotics intended for zootechnical animals (those that do not have a limit are prohibited) [7], it is increasingly desirable to reach zero residues in milk, not only from the point of view of a “One Health” logic, but also to limit technological problems during cheese-making that can lead to significant economic losses.

This study is a continuation of our recent work mentioned above [6], but in this case the multiclass detection of antibiotics in milk through modern HPLC-HRMS, was carried out after protocols of therapeutic administration, respecting the withdrawal time. This study also evaluated the reliability of screening tests in comparison with HPLC-HRMS confirmation techniques, to verify the absence or possible presence of drugs in small concentrations which, despite the observance of withdrawal time, could still interfere with the technological processes, especially regarding PDO (Protected Denomination of Origin) products. Moreover, the targeted search for the previously administered antibiotics was implemented through a non-targeted search for their metabolites, which could still be pharmacologically active and could also interfere in the cheese-making process [6]. The proposed non-targeted approach with modern instrumentation and Compound Discoverer™ software could be useful to improve the knowledge about antimicrobial metabolites in milk due to the scarce literature, as shown in Table 1.

## 2. Materials and Methods

### 2.1. Chemicals and Reagents

The 66 selected antimicrobial agents of different classes (*Enrofloxacin, Difloxacin, Danofloxacin, Levofloxacin, Lomefloxacin, Marbofloxacin, Norfloxacin, Enoxacin, Flumequine, Nadifloxacin, Oxolinic acid, Nalidixic acid, Amoxicillin, Ampicillin, Phenoxymethylpenicillin, Benzylpenicillin, Cefadroxil, Cefalexin, Cefalonium, Cefalothin, Cefazolin, Cefoperazone, Cefquinome, Cefapirin, Ceftiofur, Desfuroylceftiofur, Cloxacillin, Dicloxacillin, Benethamine penicillin, Nafcillin, Oxacillin, Piperacillin, Tylosin, Tilmicosin, Oleandomycin, Spiramycin, Neospiramycin, Kitasamycin, Josamycin, Tulathromycin, Erythromicyn A, Rifaximin, Sulfadiazine, Sulfadimethoxine, Sulfadimidine, Sulfamerazine, Sulfamethoxazole, Sulfamonomethoxine, Sulfapirydine, Sulfatiazole, Trimethoprim, Chlorotetracycline, Oxytetracycline, Tetracycline, Doxycycline, Lincomycin, Chloramphenicol, Tiamphenicol, Florfenicol, Florfenicol amine, Tiamulin, Valnemulin, Dimetridazole, Ronidazole, Tinidazole*), the internal standard, Enrofloxacin-d5, and all solvents and reagents were purchased from Merck (Darmstadt, Germany).

The Solid Phase extraction cartridges (SPE, Oasis HLB 3 mL, 60 mg) were from Waters (Milford, MA, USA). Kits used for screening tests were Delvotest^®^ SP NT plates from DSM (Heerlen, the Netherlands), the ROSA Charm QUAD1 Test from Charm Sciences Inc (Lawrence, MA, USA) and Milk Antibiotic Testing 3 in 1 Macrolides (*Erythromycin, Lincomycin, Tylosin, Tilmicosin*) 96 Tests from Shenzhen Bioeasy Biotechnology Co., Ltd. (Shenzhen, China).

### 2.2. Milk Sample Collection

A total of 141 raw bovine milk samples were collected from local farms located in the Piedmont Region, North Italy, where the majority of milk is usually used for Grana Padano PDO cheese production. The samples were all selected from dairy cows previously treated with different antimicrobial drugs (Table S1) due to medical conditions. The collection of milk was performed in accordance with the withdrawal period of all administered drugs and, in this particular case, at the 7th milking.

### 2.3. Antimicrobial Residue Analysis

As a continuation of our previous study [6], the suitability of the 3 screening tests to set up the preliminary field monitoring analyses in raw bovine milk was evaluated by comparison with the HPLC-HRMS Orbitrap confirmatory multiclass analysis.

#### 2.3.1. Screening Test Analyses

The 3 different screening kits used (Delvotest^®^ SP NT, Bioeasy—3in1 Macrolides, Charm QUAD1) and the protocols applied were well-described in previous work [6]. Briefly, quantities of 100, 200 and 300 µL for the Delvotest^®^, Bioeasy—3in1, and Charm QUAD1, respectively, were dispensed in each well and incubated at different temperatures and times (64 °C for 3 h, 40 °C for 3 min 56 °C for 5 min, respectively), and the results were evaluated immediately after incubation.

#### 2.3.2. HPLC-HRMS Confirmatory Analyses

Confirmatory analyses were performed in duplicate according to the method described in our previous work [6]. Briefly, 1 mL of raw bovine milk, spiked at 2 ng mL^−1^ with the IS, extracted with 5 mL of McIlvaine buffer (pH 4.0) and 100 μL, 20% *w/v* of Trichloroacetic acid and then defatted with hexane, was purified by HLB SPE (Hydrophilic–Lipophilic Balance for Solid Phase Extraction). Analyses were performed by an HPLC system (Thermo Fisher Scientific, San Jose, CA, USA) coupled with a Thermo Q-Exactive Orbitrap (Thermo Fisher Scientific, San Jose, CA, USA). All the mass spectrometry (MS)parameters for the full-scan acquisition (FS), combined with the data-independent acquisition (DIA), for the MS^2^ response, were also described in previous work [6].

#### 2.3.3. LC-HRMS Method Validation

The method was previously validated according to the Commission Decision 2002/657/EC guidelines [16] and SANCO/2004/2726 revision 4 [17], as reported in Chiesa et al. [6], where recovery, the decision limit (CCα) and detection capability (CCβ), and precision, in terms of intra- and inter-day repeatability, were fully assessed in compliance with the recommended tolerance ranges of the guidelines.

#### 2.3.4. Compound Discoverer Software for Expected and Non-Targeted Metabolites

The multiclass antimicrobial metabolite list with formula and parent exact mass [*m*/*z*], in Electrospray Ionization (ESI) positive acquisition mode, used for confirmatory analysis after the Compound Discoverer™ approach, and literature information are reported in Table 2.

Each positive sample was reprocessed in full scan data dependent mode (FS-dd-MS^2^) and the raw files obtained were submitted to Compound Discoverer (CD) 3.1 software (Thermo Fisher, Waltham, MA, USA), which enabled programmed identification of antibiotic metabolites. The resolving power of FS was adjusted on 70,000 FWHM at *m*/*z* 200, with the scan range of *m*/*z* 125–1000. Automatic gain control (AGC) was set at 3 × 10^6^, with an injection time of 200 ms. A targeted dd-MS^2^ analysis operated at 35,000 FWHM (*m*/*z* 200). The AGC target was programmed at 2 × 10^5^, and the maximum injection time was set at 100 ms. Fragmentation of precursors was optimized as three-stepped normalized collision energy (NCE) (20, 40 and 40 eV).

As CD was developed specially for Q-Exactive Orbitrap instrumentation, an already existing workflow, “Expected and Unknown Met ID Workflow: Find and identify both expected and unknown metabolites”, was applied with the addition of Fragment Ion Search (FISh) processing. FISh node is a CD operation segment that enables structural confidence scoring to predict fragment ions from a given parent compound. This workflow performs retention time alignment, detects expected compounds, dealkylation and dearylation products and bio-transformation products with resolution-aware isotope pattern matching, detects unknown compounds after mass defect filtering, and groups expected compounds and unknown compounds across all samples. It also proposes the elemental compositions for all unknown compounds and hides chemical backgrounds (using Blank samples).

A database of possible antibiotic metabolites was created from the features that were isolated by CD, and all revealed structures were confirmed manually using the classic method for fragment recognition.

## 3. Results and Discussion

The results of all 141 raw bovine milk samples, analyzed in duplicate, are reported in Table S1, while only the milk samples in which antimicrobial agents were found after confirmatory analysis are shown in Table 3 with the relative screening test results and information about treatments.

As can be observed, despite the fact that the withdrawal period was amply respected, with the sample collection after the seventh milking, 41 samples (29%) showed residual presence of a treatment compound. Moreover, in 9% of the total samples some compounds not indicated in the treatment protocol of the animal were detected, often also during screening tests. In particular, a major unexpected finding was that MRLs were exceeded in eight samples (20% of the positives, or 6% of the total) as reported in bold in Table 3. In this regard, in two samples, the exceeded compound (amoxicillin) was not declared amongst the treatments. As preliminarily reported in our previous study [6], also in this case a discrepancy was found between screening and confirmatory analyses and between the different screening tests used. In particular, there were 68, 27 and 20% false negatives for the Delvotest^®^ SP NT, Charm QUAD1 and Bioeasy—3in1 Macrolides, respectively, and 12, 14 and 6% false positives, respectively. The false positive and negative percentages were calculated on the basis of the appropriate screening test applied for the declared treatment. If these percentages are compared with the previous study, the false positives were comparable, whereas the false negatives showed much higher percentages in this work based on real treatments. This point is crucial because, as demonstrated before, these non-detected residues can interfere with the cheese-making process and have a negative impact on milk starter-cultures, as demonstrated during the previous microbiological analysis which checked lactic acid bacteria and the total microbial count [6]. Usually, in fact, only samples which are found to be positive by screening tests undergo confirmatory analysis. On the other hand, the non-specific characteristics of screening tests are well known and have also been reported in the literature in regard to milk [18] and other matrices [19,20]. Moreover, some discrepancies (five cases out of 41 positives, 12%) between the compounds detected by the screening tests and the confirmatory analyses are also apparent, as shown in Table 3.

**Figure 1:**
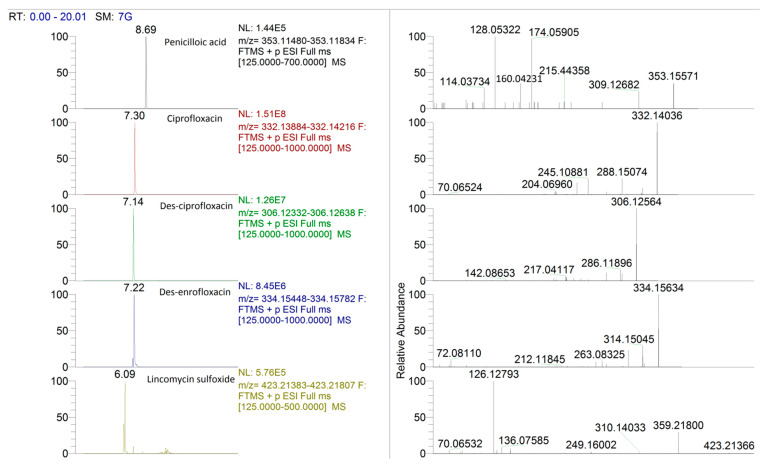
Extracted chromatograms and mass spectra of the metabolites found in our real samples.

Regarding the non-targeted analysis performed by Compound Discoverer, the presence of metabolites was also then confirmed by the targeted list and assessed afterwards. Extracted chromatograms and mass spectra of the metabolites found in the real samples are presented in Figure 1.

In particular, metabolites were found in all samples with the presence of enrofloxacin and lincomycin, with three metabolites (ciprofloxacin, des-ciprofloxacin and des-enrofloxacin) for the detected quinolone and lincomycin sulfoxide for the lincosamide.

**Figure 2:**
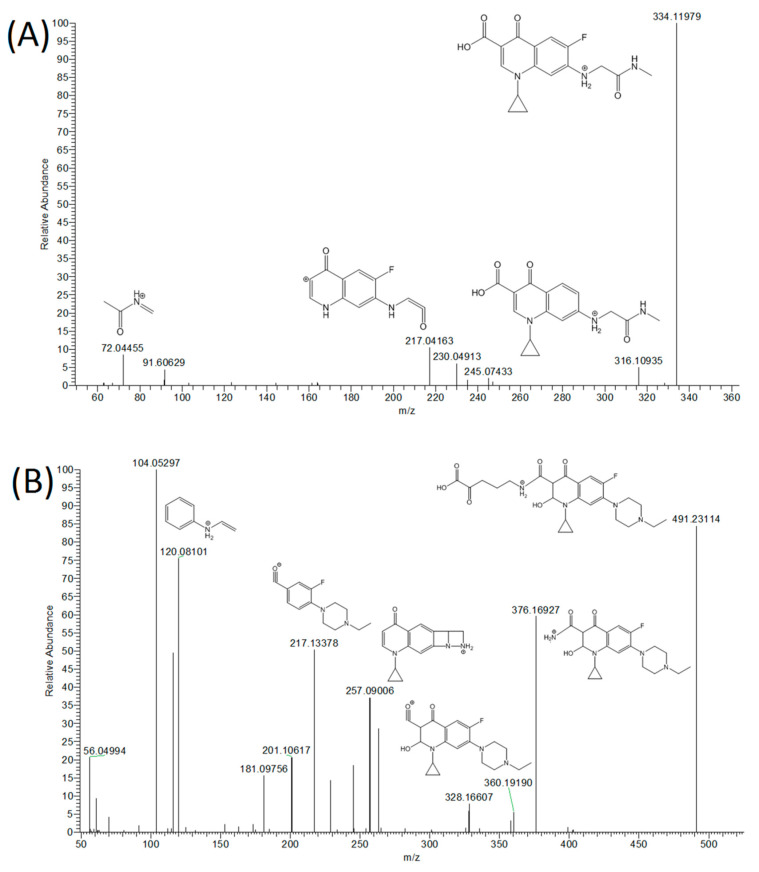
Mass spectral data, proposed structures and MS2 spectrum for two new enrofloxacin metabolites. (**A**) ENRO-N-methylacetamide and (**B**) ENRO-ornithine.

Enrofloxacin, according to the literature [12,21], is extensively metabolized into ciprofloxacin and other minor metabolites, with the former still retaining antimicrobial activity. Two new metabolites that had not been reported in the literature were tentatively identified (Table 3, Figure 2).

The ion with *m*/*z* 334.1198 including its MS^2^ spectrum can be attributed to a compound resulting from the break-up of the piperazine ring followed by introduction of the keto function in α-position respect to the terminal secondary amino group. For this reason, this compound, entitled 1-cyclopropyl-6-fluoro-7-((2-(methylamino)-2-oxoethyl amino)-4-oxo-1,4-dihydroquinoline-3-carboxylic acid) according to IUPAC (International Union of Pure and Applied Chemistry) nomenclature is abbreviated as ENRO-N-methylacetamide and can be considered a phase I metabolite. The second metabolite (5-(1-cyclopropyl-7-(4-ethylpiperazin-1-yl)-6-fluoro-2-hydroxy-4-oxo-1,2,3,4-tetrahydroquinoline-3-carboxamido)-2-oxopentanoic acid) named ENRO-ornithine (*m*/*z* = 491.2311) proved to be essential because its absolute intensity was similar to enrofloxacin itself. It is formed during a three-step metabolomic transformation that includes water addition to the double bond of the quinoline ring and conjugation with ornithine (phase II) that is further modified by oxidative deamination. Some metabolites of Enrofloxacin are cleared from plasma to milk after experiencing conjugation with an amino acid, for example lysine [22], but ornithine conjugate has not been reported so far. In particular, ciprofloxacin (198.49 ng mL^−1^), des-ciprofloxacin (14.34 ng mL^−1^), des-enrofloxacin (6.05 ng mL^−1^) ENRO-N-methylacetamide (1.90 ng mL^−1^) and ENRO-ornithine (27.50 ng mL^−1^) were found in one sample treated with enrofloxacin, in which the parent drug was found at a higher concentration (25.50 ng mL^−1^, Table 3). The detection of ciprofloxacin (3.80 ng mL^−1^) and of the new putative proposed metabolite, ENRO-ornithine (23.35 ng mL^−1^), in one sample not declared for enrofloxacin treatment, in which the parent drug was detected at 0.20 ng mL^−1^ (Table 3), was an important finding.

Lincomycin, too, is extensively metabolized [21] with three major metabolites (lincomycin sulfoxide, N-desmethyl linomycin, N-desmethyl lincomycin sulfoxide). Compared to the parent compound, both N-desmethyl and lincomycin sulfoxide have 15 to 100 times less antimicrobiological activity than lincomycin. There was no evidence that the remaining metabolites have any antimicrobiological activity [23]. In the samples treated with lincomycin, only lincomycin sulfoxide was found, in the range of 0.77–1.22 ng mL^−1^, in the milk where the parent drug was present. In particular, this metabolite was found in two samples not declared for lincomycin administration, providing proof of the real presence of the parent drugs (Table 3, lincomycin 35.61 and lincomycin 0.53 ng mL^−1^). In the samples in which lincomycin was below 0.50 ng mL^−1^, the metabolite was < CCα.

In the absence of all detected metabolite standards, concentration quantitation was performed using the parent drug matrix calibration curve. Moreover, penicilloic acid was also detected in one sample from an animal previously treated with both lincomycin and benzylpenicillin, where the parent drug found (amoxicillin, conc. 50.59 ng mL^−1^), not only was not declared, but also exceeded the MRL. This is particularly interesting due to the fact that the withdrawal period was fully respected and the samples were collected at the seventh milking. This finding could be proof of a non-declared treatment even if penicilloic acid could also derive from benzylpenicillin [21]. According to the information presented in the literature [21], both amoxicillin and belzylpenicillin are not extensively metabolized but the major metabolite, penicilloic acid (accounting for 10–25% of total parent residue), is also considered to have allergic potential [24].

In the light of the results obtained, the list of treatments administered to animals was important in the search for both parental drugs and any possible metabolites due to the high number of false negative and positive percentages of screening tests. Regarding the presence of metabolites, not only their toxicological potential but also their negative impact on dairy technological processes must be considered a matter for concern.

## 4. Conclusions

Antibiotic residues in milk are a serious health and technological problem in dairy processes. Multi-class analysis after different therapeutical administrations, even if carried out on samples that largely respected withdrawal times, highlighted the presence of parent drugs in 29% of cases and revealed undeclared treatments, not only through the presence of the parent compound, but also of its metabolites.

Moreover, this last point can be supported by the discovery of new proposed metabolites for enrofloxacin, through the new Compound Discoverer software approach for expected and non-targeted metabolites. In light of this evidence, it must be borne in mind that metabolites, some of which are pharmacologically active, may also pose a risk to the consumer and the dairy industry.

Lastly, as a result of information about the treatments, it was possible to verify more accurately, through confirmatory analyses, the reliability of screening tests, highlighting their high non-specificity and limited usefulness under field conditions.

## Figures and Tables

**Table 1:** State of the art regarding antimicrobial metabolites and degradation products in milk.

Reference	Analytes	Metabolites and Degradation Products	Matrix	Extraction Technique	Instrumental Analysis	Detected Concentration Range (ng mL^−1^)
[8]	Lincomycin (A)	Undefined metabolites	Milk	L/L Extraction, deproteinization	HPLC-UV	No application
[9]	Enrofloxacin	Ciprofloxacin	Goat milk	SPE	HPLC-DAD	No application
[10]	Oxytetracycline Tetracycline Chlortetracycline Doxycycline Demeclocycline Methacycline Minocycline	4-epioxytetracycline 4-epitetracycline 4-epichlortetracycline	Milk	HLB SPE	LC-MS/MS	No application
[11]	Amoxicillin Penicillin G	Amoxicilloic acid Amoxicillin diketopiperazine-2′,5′-dione Benzylpenicilloic acid Benzylpenilloic acid Benzylpenillic acid	Milk	Defatting, L/L Extraction MICRO PES filtration	UHPLC-MS/MS	Benzylpenicilloic acid (N.D-446) Benzylpenilloic acid (9–867)
[12]	Cephapirin Enrofloxacin Sulfamethazine	Desacetylcephapirin Ciprofloxacin Des-ENR Pefloxacin desethylene-CIP N4-acetyl metabolite lactose conjugate	Milk	SPE	Q-TOF LC-MS	No application
[13]	Cephapirin Cefquinome Ceftiofur Cefacetrile Cefalonium Cefalexin Cefazolin Cefoperazone Cefradine Cefotaxime	Desacetylcephapirin	Milk	SPE	LC-MS/MS	Cefalexin (12.7–166.2)
[14]	Amoxicillin Penicillin G Cephapirin Ceftiofur	Penilloic acid Amoxicillin diketopiperazine-2′,5′-dione Amoxicilloic acid PENG-1-5 PENG-1 (benzylpenilloic acid) PENG-4 (benzylpenicilloic acid) (PIR-1 to PIR-10) PIR-4 (cephapirin lactone) PIR-5 (desacetylcephapirin) PIR-7 (methoxy desacetylcephapirin) PIR-8 (desacetylcephapirin methyl ester) (TIO-1 to TIO-5) TIO-2 (deacetylcefotaxime)	Milk	SPE	LC–HRMS	No application
[15]	Sarafloxacin Gatifloxacin Ofloxacin Enrofloxacin Lomefloxacin Ciprofloxacin Enoxacin Norfloxacin Sulfadiazine Sulfamethoxazole Sulfamerazine Sulfamethazine Sulfameter	N4-Acetylsulfadiazine N4-Acetylmethoxazole N4-Acetylsulfamerazine N4-Acetylsulfamethazine	Milk	HLB SPE	UPLC-MS/MS	Ofloxacin (13.1–36) Enrofloxacin (14.2–24) Ciprofloxacin (14–44) N4-Acetylmethoxazole (8–29) N4-Acetylsulfamerazine (11–30)

**Table 2:** Multiclass antimicrobial metabolite list with formula and exact mass in ESI positive acquisition mode [*m*/*z*] used for confirmatory analysis after Compound Discoverer™ approach and literature information.

Parent Exact Mass [*m*/*z*]	Formula	Metabolite
382.05259	C_15_H_15_N_3_O_5_S_2_	Desacetylcefapirin
430.03081	C_14_H_15_N_5_O_5_S_3_	Desfuroylceftiofur
332.14050	C_17_H_18_FN_3_O_3_	Ciprofloxacin
334.15615	C_17_H_20_FN_3_O_3_	Pefloxacin
334.11979	C_16_H_16_FN_3_O_4_	ENRO-N-methylacetamide
334.15615	C_17_H_20_FN_3_O_3_	Des-Enrofloxacin
306.12485	C_15_H_16_FN_3_O_3_	Des-Ciprofloxacin
346.11976	C_17_H_16_FN_3_O_4_	Oxo-Ciprofloxacin
491.23004	C_24_H_31_FN_4_O_6_	ENRO-ornithine
423.21595	C_18_H_34_N_2_O_7_S	Lincomycin sulfoxide
408.19247	C_17_H_31_N_2_O_7_S	Desmethyl lincomycin sulfoxide
393.20538	C_17_H_32_N_2_O_6_S	Desmethyl lincomycin
224.11286	C_8_H_17_NO_6_	Lincosamine
349.08527	C_16_H_16_N_2_O_5_S	Amoxicillin desaminated
340.13255	C_15_H_21_N_3_O_4_S	Amoxicillin penilloic acid
366.11182	C_16_H_19_N_3_O_5_S	Diketopiperazine amoxicillin
384.12238	C_16_H_21_N_3_O_6_S	Amoxicillin penicilloic acid
397.13021	C_17_H_22_N_3_O_6_S	Amoxicilloic acid methyl ester
515.15950	C_24_H_26_N_4_O_7_S	4-Hydroxyphenylglycyl amoxicillin
382.10673	C_16_H_19_N_3_O_6_S	Amoxicillin-S-oxide
208.08424	C_10_H_11_N_2_O_3_	Amoxicillin penicilloaldehyde
285.05397	C_11_H_12_N_2_O_5_S	Amoxicillin penaldic acid
333.09035	C_16_H_16_N_2_O_4_S	Ampicillin desaminated
368.12747	C_16_H_21_N_3_O_5_S	Ampicillin penicilloic acid
324.13764	C_15_H_21_N_3_O_3_S	Ampicillin penilloic acid
396.12238	C_17_H_21_N_3_O_6_S	Ampicilloic acid methyl ester
483.16967	C_24_H_26_N_4_O_5_S	D-phenylglycylampicillin
353.11657	C_16_H_20_N_2_O_5_S	Penicilloic acid
293.07029	C_12_H_12_N_4_O_3_S	N-acetylsufadiazine
353.09142	C_14_H_16_N_4_O_5_S	N-Acetylsulfadimethoxine

**Table 3:** Confirmed samples with antibiotic presence, relative screening test results and information about treatments.

	Screening Tests			Confirmation Analysis (HPLC-HRMS)	Treatments
N°	Delvotest SP NT	Charm QUAD1	Bioeasy 3in1	Conc. (ng mL^−1^ ± SD)	
1	N	N	n.a	oxytetracycline (2.63 ± 0.03)	cefquinome, oxytetracycline
5	N	N	n.a	oxytetracycline (0.22 ± 0.02)	oxytetracycline
6	N	N	n.a	oxytetracycline (0.57 ± 0.03)	oxytetracycline
7	N	n.a	N	lincomycin (0.46 ± 0.02)	lincomycin
8	N	P sulfonamide	n.a	sulfadiazine (5.73 ± 0.04)	sulfadiazine
9	N	P sulfonamide	n.a	sulfadiazine (6.92 ± 0.03)	sulfadiazine
11	P	n.a	N	lincomycin (0.43 ± 0.02)	lincomycin
13	N	N	P lincomycin	lincomycin (2.78 ± 0.05)	lincomycin, benzylpenicillin
15	N	P tetracycline	n.a	oxytetracycline (14.57 ± 0.06)	oxytetracycline
16	P	P β-lactam	n.a	**cefalonium (51.01 ± 0.07)**	cefalonium
22	N	N	n.a	**amoxicillin (52.57 ± 0.06)**	amoxicillin, benzylpenicillin
29	N	P quinolone	n.a	enrofloxacin (0.20 ± 0.02)	oxytetracycline
32	N	N	N	lincomycin (0.41 ± 0.03)	lincomycin, sulfadiazine
33	P	P β-lactam	n.a	**cloxacillin (250.69 ± 0.07)**	cloxacillin
37	P	P β-lactam	n.a	cefquinome (<CCβ)	marbofloxacin
38	N	N	n.a	oxytetracycline (2.65 ± 0.04)	oxytetracycline
39	N	n.a	P lincomycin	lincomycin (2.26 ± 0.04)	lincomycin
47	N	P sulfonamide	n.a	sulfadiazine (10.42 ± 0.06)	Other drugs
48	N	P sulfonamide	n.a	sulfadiazine (6.45 ± 0.07)	marbofloxacin
50	N	n.a	N	lincomycin (0.36 ± 0.04)	lincomycin
51	P	P β-lactam	P lincomycin	lincomycin (0.53 ± 0.03), **cloxacillin (32.96 ± 0.08)**	cloxacillin
52	N	N	P lincomycin	lincomycin (35.61 ± 0.07)	cefquinome
54	N	P tetracycline	n.a	oxytetracycline (15.29 ± 0.05)	oxytetracycline
59	P	P quinolone	n.a	enrofloxacin (25.50 ± 0.04)	enrofloxacin, benzylpenicillin
61	N	P tetracycline	n.a	oxytetracycline (17.08 ± 0.08)	cefquinome, oxytetracycline
64	N	P tetracycline	n.a	oxytetracycline (5.62 ± 0.06)	oxytetracycline
66	N	P tetracycline	n.a	sulfadiazine (0.80 ± 0.03)	oxytetracycline
69	N	P quinolone	N	lincomycin (1.13 ± 0.04)	lincomycin
76	N	P quinolone	N	**amoxicillin (50.59 ± 0.06)**	lincomycin, benzylpenicillin
79	N	P β-lactam	n.a	oxytetracycline (22.37 ± 0.05)	benzylpenicillin
80	P	P β-lactam	N	lincomycin (0.95 ± 0.03)	lincomycin
81	P	P β-lactam	n.a	**cloxacillin (1281.95 ± 0.16)**	cloxacillin
82	N	P tetracycline	n.a	oxytetracycline (42.87± 0.09)	cefquinome
96	N	N	P lincomycin	lincomycin (1.65 ± 0.06)	lincomycin, sulfadiazine
101	P	P β-lactam	n.a	cloxacillin (322.39 ± 0.12)	cloxacillin
121	P	N	n.a	cefalonium (7.52 ± 0.09)	cefalonium
125	N	P tetracycline	n.a	oxytetracycline (0.68 ± 0.07)	oxytetracycline
127	P	P sulfonamide	P lincomycin	**amoxicillin (18.35 ± 0.11)**, sulfadimethoxine (< CCβ)	lincomycin, benzylpenicillin
132	P	P β-lactam	n.a	lincomycin (0.30 ± 0.05)	cloxacillin
133	P	P β-lactam	n.a	**cloxacillin (83.63 ± 0.11)**	cloxacillin
134	N	N	n.a	oxytetracycline (2.52 ± 0.08)	cefquinome

N = negative, P = positive, n.a = Not applicable. In bold—only concentrations exceeding MRLs.

## Supplementary Materials

The following are available online at https://www.mdpi.com/2304-8158/10/3/544/s1, Table S1: Raw data about screening tests and confirmatory analyses of 141 bovine raw milk samples, with related treatment information.
